# Nursing staff adherence to guidelines on nutritional management for critically ill patients with cancer: A service evaluation

**DOI:** 10.1111/nicc.13062

**Published:** 2024-03-20

**Authors:** Marie Parsons

**Affiliations:** ^1^ Florence Nightingale Faculty of Nursing, Midwifery and Palliative Care King's College London London UK; ^2^ The School of Biological Sciences The University of Edinburgh Edinburgh United Kingdom of Great Britain and Northern Ireland

**Keywords:** critical care, enteral feeding, gastric residual volumes, oncology nursing

## Abstract

**Background:**

Critically ill patients with cancer are at high risk of developing malnutrition, negatively affecting their outcome.

**Aim:**

To critically analyse nursing staff's adherence to nutrition management guidelines for critically unwell patients with cancer and identify barriers which prevent this. Two areas of nutrition management were evaluated: early initiation (<48 h from admission) of enteral nutrition (EN) and continuation of EN without interruption.

**Study Design:**

A retrospective data analysis was performed on mechanically ventilated adult patients admitted to a single cancer centre. Data from electronic patient records (EPR) were collected. Health care professionals' (HCP) documentation was analysed, and a nursing staff focus group (*n* = 5) was undertaken.

**Results:**

Sixty‐four patient records were included. Early EN was not administered in 67% (*n* = 43) of cases. The reasons for the three longest interruptions to EN feed were as follows: delays in EN tube insertion, gastric residual volumes (GRVs) less than the recommended feed discontinuation threshold and endotracheal intubation. Four main themes relating to barriers to practice were identified from the focus group data analysis: HCPs' approach towards nutrition management, the patient's physiological condition and stability, multi‐disciplinary team (MDT) communication and guidance on nutrition management, and practical issues with patient care.

**Conclusions:**

Multi‐disciplinary communication difficulties, lack of clear guidelines and inadequate awareness of the importance of nutrition for critically ill patients with cancer were barriers identified preventing optimal nutrition management.

**Relevance to Clinical Practice:**

Nursing education is fundamental to help break down the barriers to practice which prevent critically ill patients from receiving optimal nutrition management.

**What is known about the topic**

- Critically ill patients with cancer are at high risk of developing malnutrition which increases risk of mortality.
- Guidelines recommend critically ill patients who are unable to consume an oral diet and those who do not show signs of intolerance or uncontrolled shock should receive enteral nutrition within the first 48 h of admission. This should be administered continuously with minimal interruption to improve outcomes.
- Previous research has shown barriers occur in practice, preventing health care professionals' adherence to nutritional guidelines.

**What this paper adds**

- This paper focuses specifically on optimal nutrition management for critically ill patients with cancer.
- It contributes to research into nursing staff adherence to nutrition management guidelines.
- Multidisciplinary team communication difficulties, lack of clear guidelines and inadequate awareness of the importance of nutrition for critically ill patients with cancer were barriers identified preventing optimal nutrition management.

## 1 INTRODUCTION

Critically ill patients with cancer are at high risk of developing malnutrition which increases risk of mortality.^1^, ^2^, ^3^ Critical illness is associated with a hyper‐catabolic state leading to the breakdown of lean body mass.^4^, ^5^ This causes complications and lengthens recovery time.^1^, ^4^, ^6^ Providing timely and tailored nutrition therapy is paramount in meeting these increased metabolic needs and improving patient outcomes.^4^, ^5^, ^7^

## 2 BACKGROUND

### 2.1 Early enteral nutrition initiation over delayed enteral nutrition in critically ill patients

During critical illness, loss of gut function can lead to adverse changes in intestinal tract permeability, increasing exposure to bacteria and, therefore, increasing systemic infection risk.^5^, ^8^ It has been reported that enteral nutrition (EN) preserves the gastrointestinal and pulmonary mucosal barrier, modulates the inflammatory responses and maintains immune function.^4^, ^5^ Therefore, guidelines report early EN (within 48 h of admission) should be considered on par with other therapies supporting organ function in critical illness.^7^

The Society of Critical Care Medicine and American Society for Parenteral and Enteral Nutrition (SCCM/ASPEN)^5^ found a reduction in infectious morbidity (*p* = .01) and mortality (*p* = .05) in patients receiving early EN compared with delayed EN.^5^ Similarly, The European Society for Clinical Nutrition and Metabolism (ESPEN) found a significant reduction in infectious complications (*p* < .03) with early EN.^7^ Recent retrospective and prospective studies support the use of early EN^8^, ^9^ but high‐quality randomized controlled trials (RCTs) are needed to provide a more reliable insight into this.

The ESPEN (2018) and the European Society of Intensive Care Medicine (ESCIM 2017) guidelines suggest withholding EN when a patient is experiencing uncontrolled shock, uncontrolled hypoxaemia and acidosis and uncontrolled upper GI bleeding. It should also be withheld if gastric aspirates of >500 mL/6 h are produced, there is evidence of bowel ischemia, bowel obstruction, abdominal compartment syndrome or a high‐output fistula without distal feeding access.^7^, ^10^

### 2.2 Early EN over parenteral nutrition in critically ill patients

The benefits of EN compared with parenteral nutrition (PN) is of much debate in the literature. SCCM/ASPEN guidelines recommend PN only when there are contradictions to EN administration.^5^ They found infectious morbidities and intensive care unit (ICU) length‐of‐stay were significantly reduced in enterally fed patients compared with PN (*p* < .00001 and *p* = .0007 respectively). ESPEN guidelines concur with this^7^ despite incorporating the two recent, multi‐centred trials; CALORIES^11^ and NUTRIREA‐2^12^ in their meta‐analysis. Controversially, the CALORIES trial found no significant difference in treated infectious complications (*p* = .72) or 30‐day mortality (*p* = .57) between the two feeding regimes and therefore challenged views on the importance of EN.^13^ It has been criticized, however, that a reduced intake of macronutrients administered in both arms may have led to these results.^14^, ^15^ The short trial duration may have also restricted the full benefits of EN.^14^ Similarly, the NUTRIREA‐2 trial showed no significant difference in the incidence of ICU‐acquired infections between PN and EN and reported a significantly higher prevalence of bowel ischemia (*p* = .007) in EN fed patients. Because of a lack of blinding, however, there was risk of bias in reporting GI dysfunction in this study.^12^ It is expected as practice progresses with improvements in glycaemic control, improved line care and use of protocols, PN is likely to be associated with fewer infectious complications in future.^5^, ^14^

### 2.3 EN administration without interruption

Interruption of continuous EN feed in preparation for therapeutic procedures or because of perceived feeding intolerance, for example, is common in practice.^2^, ^16^ EN interruption is associated with substantial calorie deficits contributing to an increased risk of malnutrition.^17^ SCCM/ASPEN guidelines report patients only receive approximately 80% of prescribed EN and interruption occurs in >85% of patients for an average of 8%–20% of infusion time.^5^ Guidelines for EN administration in critically ill patients advise to minimize interruptions but do not identify a standard protocol in order to do this.^5^, ^17^

This service evaluation is the first to focus specifically on adherence to guidelines regarding the optimal nutrition management for critically unwell patients with cancer. Guidelines recommending the early initiation of EN and continuation of EN without interruption were focused on based on their significance in affecting patient outcomes, their relevance to nursing practice and the strength of their supporting evidence.^5^, ^7^, ^10^ Exposing barriers to practice which prevent guideline adherence is a step towards improving nutritional management in this centre.^2^ It aims to contribute some valuable information on nutrition management for this patient group.

## 3 AIM

This small‐scale preliminary service evaluation aimed to critically analyse nursing staff adherence to nutrition management guidelines for critically ill patients with cancer and identify the barriers which prevent this. Two components of nutrition management were the focused upon: early initiation of EN and continuation of EN without interruption.

## 4 DESIGN AND METHODS

### 4.1 Study design

A mixed‐methods designed was used. This service evaluation took place within an ICU in an NHS Trust specializing in cancer care in the United Kingdom. Retrospective data from Electronic Patient Records (EPR) was collected and analysed. Additionally, data were collected from a nursing staff focus group on factors which impact nutrition management in the ICU setting.

### 4.2 Quantitative data collection

An ideal sample size of 369 patients was calculated using the Sample Size Calculation for Population Proportion Estimation.^18^, ^19^, ^20^ A coded randomized list of mechanically ventilated patients admitted to ICU within the last year was generated by the Trust's Information System Manager from the EPR system. This list was then screened based on the inclusion and exclusion criteria (Table 1). Mechanically ventilated patients are reliant on nutritional support as oral intake is not feasible; therefore, those patients intubated within the first 24 h of admission were included. This allowed an adequate window of time for decisions around EN feed commencement to be put into place, within 48 h of admission. Patients able to eat an oral diet within the first 48 h of admission were excluded as were those where EN was inappropriate according to guidelines. Medical records were scrutinized, and notes were taken for each record.

**TABLE 1:** Inclusion and exclusion criteria for quantitative data collection.

Inclusion	Exclusion
Patients intubated within the first 24 h of ICU admission (an indicator they need artificial feeding) Elective and emergency admission	Patients started on an oral diet EN delayed because of valid clinical reasons which are as follows: Where shock is uncontrolled and hemodynamic and tissue perfusion goals are not reached despite vasopressors/inotropes or fluid administration. Where unstable doses of vasoactive drugs are used^5^ In uncontrolled life threatening hypoxemic, hypercapnic or acidotic circumstances In patients suffering from active upper GI bleed In patients with overt bowel ischemia In patients with high‐output intestinal fistula if reliable feeding access distal to fistula not achievable In patients with abdominal compartment syndrome gastric aspirate volume >500 mL/6 h. Gastric contents can be aspirated from the stomach via the nasogastric (NG) tube. Any contents measuring more than 500 mL every 6 h would be considered too high and indicates poor gastric emptying^7^

Abbreviations: EN, enteral nutrition; GI, gastrointestinal; ICU, intensive care unit.

### 4.3 Qualitative data collection

Nursing staff were recruited on a voluntary basis and selected using positive sampling. This was based on whether they were regularly employed on the unit and had experience working with intubated patients requiring enteral nutrition.^21^, ^22^, ^23^ This was to ensure those involved had an awareness of local policies and procedures. Following a recruitment email all staff who responded were then subsequently involved. Written and verbal consent was gained from participants. Information regarding the learning objectives, matters of confidentiality and the time commitment involved was provided. Their anonymity would be upheld throughout the process. Five participants were involved: three senior nurses and two junior nurses, providing an equal spread of experience within the group. This small group permitted a relaxed discussion where all members were able to provide insights into their practice.

#### 4.3.1 Measures

Demographic data and reason for ICU admission were obtained from patients' records. To measure if early EN was administered appropriately and without interruption as per guidelines, information on the following was extracted from data: route and type of nutrition given, time taken to initiate feed post admission, reasons why EN was not initiated or was delayed and number of occasions and length of time EN, if started, was disrupted. Records were analysed to find clinical reasons why EN may have been delayed or omitted as listed in the exclusion criteria.

When the focus group stimulus was open, non‐leading questions were used as a tool to guide the discussion. Notes were made in the focus group by an observer to capture body language and other behavioural factors.^24^ The discussion was recorded using a Trust Dictaphone and transcribed verbatim independently.

#### 4.3.2 Analysis

For each aspect of nutrition management focused on, descriptive analysis of the quantitative data was performed to illustrate results.

Qualitative data from the focus group were analysed using the ‘Framework Method’, a structured, systematic approach which identifies similarities and differences and relationships within the data, and subsequent conclusions clustered around themes.^25^, ^26^ Areas of significance in the transcript were highlighted, and codes were then assigned to these areas of text. Descriptions of each code were developed, and the coding process was refined if multiple codes were describing the same concept in a different way. Conceptually related codes were grouped together to form overarching themes.^25^ Extracted transcript data were charted comprising of a row per participant and a column per code for each theme. This framework allows the researcher to compare data across multiple focus groups^25^; however, in this instance, a single focus group was carried out because of issues with participant recruitment.

#### 4.3.3 Ethical considerations

Ethical approval was not needed for this service evaluation in accordance with the NHS Health Research Authority.^27^ The Trust's Committee for Clinical Research reviewed and approved the proposal for the service evaluation. Patients' privacy and confidentiality was upheld. Data were made anonymous and stored in a password protected document on the hospital's server.

## 5 RESULTS

### 5.1 Quantitative data

A total of 590 patients were identified requiring mechanical ventilation between 2017 and 2018. These were presented to the author coded and randomized to reduce bias. Because of time constraints 211 of these were screened, and from these, 64 records were included according to the inclusion and exclusion criteria. Out of the studied population (*n* = 64), 59% (*n* = 38) of males and 41% (*n* = 26) females were included. The mean age of patients included was 63 ± 13 years. Out of all cases studied, 73% (*n* = 47) were admitted for elective surgery and 27% (*n* = 17) for medical emergencies. Admittance following upper and lower gastrointestinal (GI) elective surgery made up most cases (51% [*n* = 33]).

**FIGURE 1:**
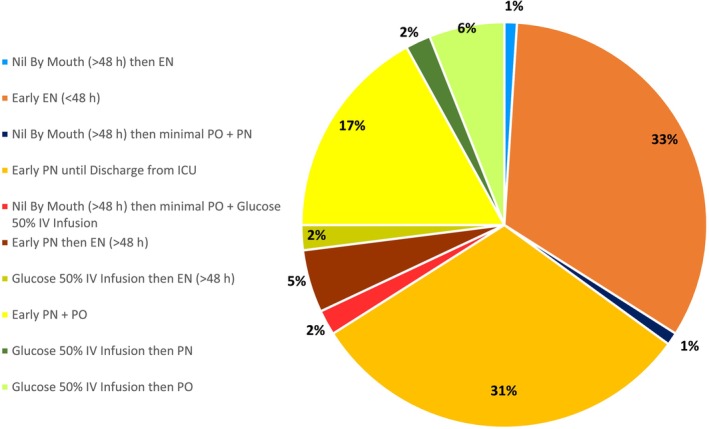
Feeding regimes provided in all cases included (*n* = 64). EN, enteral nutrition; ICU, intensive care unit; IV, intravenous; PN, parenteral nutrition; PO, per oral/taken orally.

Of the 64 cases included, 67% (*n* = 43) did not receive EN within the first 48 h of admission to ICU despite it being deemed appropriate following a case‐by‐case analysis with reference to guidelines.^5^, ^7^, ^10^ The feeding regimes given in all cases are displayed in Figure 1. A total of 97% of patients (*n* = 62) were admitted in the ICU with a nasogastric tube (NG) in situ. The remaining 3% (*n* = 2) did not have any feeding tubes in situ on admission. No patients had a nasojejunal tube (NJ) or jejunostomy inserted at any point during their stay.

**FIGURE 2:**
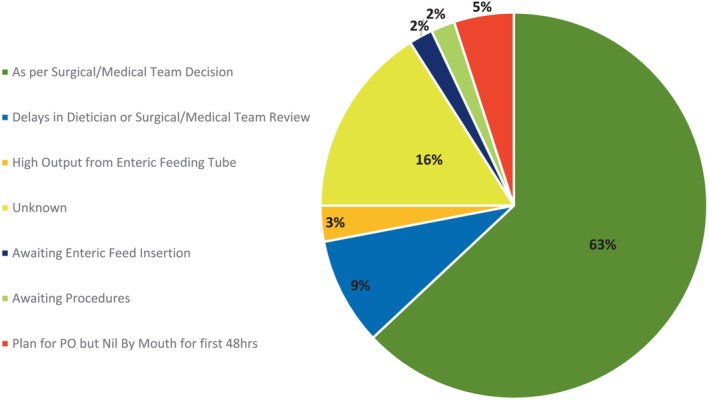
Reasons found for delayed or omitted enteral nutrition (EN) from health care professionals documentation in all cases who did not receive early EN (*n* = 43). PO, per oral/taken orally.

Of all patients who did not receive early EN (*n* = 43), it was documented in 63% (*n* = 27) of these cases that EN was delayed or omitted because of ‘surgical/medical team preference’ (Figure 2). On analysis of all upper GI elective surgical patients (*n* = 15), 87% (*n* = 13) received PN ‘as per a surgical team decision’, according to patient documentation. Similarly, 78% (*n* = 14) of all lower GI elective surgical cases (*n* = 18) were administered PN within the first 48 h of admission ‘as per surgical team decision’, according to patient documentation.

**FIGURE 3:**
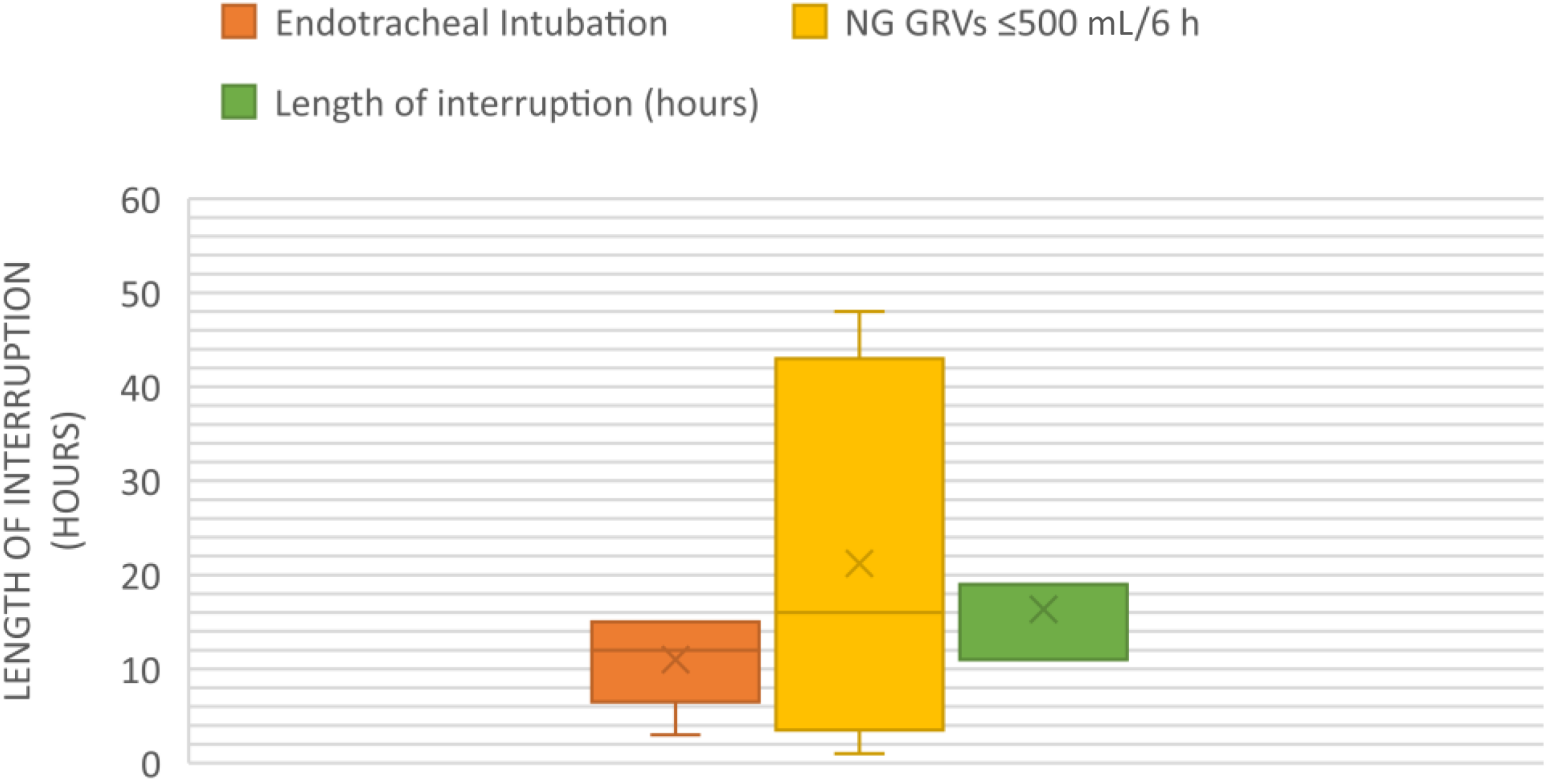
Three longest enteral nutrition interruptions (h)—median values and interquartile ranges (IQR). GRVs, gastric residual volumes; NG, naso gastric.

Of the 21 cases in which continuous EN was administered (regardless of whether there was a delay in initiation), the number of occasions and length of time it was interrupted were recorded (Figure 3). A total of 106 interruptions were identified. These were grouped under two main categories: physiological concerns and clinical interventions, with 19 sub‐categories. The reasons for the three longest EN interruptions (median length hours; interquartile range) were as follows: Awaiting NG reinsertion (19 h; 11–19 h), NG GRVs of ≤500 mL/6 h (despite guidelines recommending interruption of feed once GRVs reach ≥500 mL/6 h, indicating poor compliance with guidelines) (16 h; 3.5–43 h) and endotracheal intubation (12 h; 6.5–15 h). Interruption of feed because of GRVs ≥500 mL/6 h as per guidelines did also occur but only caused interruptions for a median length of 4 h (interquartile range [IQR]: 3–32 h).

### 5.2 Qualitative data

The final analytical framework of the focus group consisted of 41 codes separated into four themes.

### 5.3 Theme 1: HCPs' approach towards nutrition management

Participants discussed the influence surgical team decisions have on post‐operative nutrition management. The patient's condition and type of surgery undertaken impact this decision but the surgeons themselves may have individual preferences for feeding regimes. One participant explains:

…*it depends on the surgeons. One surgeon will prefer total parenteral nutrition via the central line…, others will put in a jejunostomy for feed. It all depends on the surgeries and the surgeons' preferences. Mainly we see PN rather than enteral feed* (Participant 1, Lines 17–23).

These decisions may not necessarily follow specific guidelines or protocols or if they do, nursing staff were not always aware of the rationale behind them. The participants also reported that nutrition is not always considered as a main priority in the ICU setting and inferior to other organ support therapies:

*I think in general nutrition comes at the bottom of the priority…and it is because it's not a critical thing* (Participant 3, Line 185–186).

### 5.4 Theme 2: A Patient's physiological condition and stability

Contraindications to providing EN were discussed. The type of surgery carried out was thought to influence the nutrition management for that patient:

*…if you have a patient with oesophageal cancer or head and neck cancer their nutritional requirements will be higher because of the cancer itself. They are dehydrated, they are cachectic, so they need more nutrition* (Participant 1 Lines 33–36).

Different protocols for feeding, including those which used PN as standard, were reported to be followed post operatively depending on the type of surgery:

*After lower GI [surgery] they don't want to stimulate the gut, so they start on PN* (Participant 1, Line 75–76).

### 5.5 Theme 3: MDT communication and guidance on nutrition management

The reliance on the dietician's expertise leading to problems in their absence or when communication links are missed, was discussed. Communication breakdown between specialist teams and the ICU medical team regarding the preferred nutrition method was reported to lead to delays in its initiation.

*…they are started on emergency PN because…there's not been the discussion between the surgical team and the ICU team of what's going to happen with the feed…we haven't got that direction…they go onto glucose 50% until the team makes that decision* (Participant 2, Lines 46–50).

Further delays can also arise on nights and weekends. Specific post‐operative protocols, used to guide the ICU team in feeding regime decisions in these instances, were reported to improve delays.

Issues with protocols for GI intolerance assessment were reported. Discrepancies around measuring GRVs have been found to lead to feed being paused unnecessarily in apprehension of poor gastric emptying:

*I think it varies a lot because some people discard [feed aspirate], others may return 150mls, others will return 200mls…* (Participant 1, Lines 126–128).

### 5.6 Theme 4: Practical issues with patient care

Practical issues such as feeding tube migration leading to feed suspension was highlighted. Issues around the appropriateness of the feeding device inserted was also discussed. For example, following GI surgery drainage tubes rather than feeding tubes may be inserted:

*…they can just directly insert [the enteric feeding tube] down in surgery but they tend to put the Ryles tube in which is not compatible for our feed, and we shouldn't be using…it's only for gastric aspirates* (Participant 3, Lines 433–435).

Insertion of a device during surgery is favoured, allowing for EN to be started without delay at the most suitable opportunity.

## 6 DISCUSSION

Two different components of nutrition management were included based on their significance in affecting patient outcomes and their relevance to nursing practice. Guidelines recommend patients who are unable to consume an oral diet and those who do not show signs of intolerance or uncontrolled shock, should receive EN within the first 48 h of ICU admission, in order to improve outcomes.^5^, ^10^ This should be administered continuously with minimal interruption. Only partial compliance to guidelines with respect to these recommendations was found in this centre. Following data analysis, the barriers which prevent nursing staff adhering to guidelines are as follows:

### 6.1 HCPs' approach towards nutrition management

The focus group highlighted that nutrition management may be considered by HCPs inferior to other organ support therapies in ICU which may contribute to poor guideline adherence. This may be because it does not feature in the ABCDE systematic approach, imperative when assessing a critically unwell patient, making it a lower priority.^28^

The focus group revealed the decision to administer a certain therapy may ultimately be dependent on a consultant's or surgeon's preference. Of the 64 cases analysed, 52% (*n* = 33) were upper and lower GI elective surgical patients. Of these 33 cases, 82% (*n* = 27) received PN instead of early EN ‘as per surgical team decision’ according to documentation. This decision may be influenced by two factors; the physiological instability of the patient, which in this case was not detected via patient record analysis, or by alternative evidence associated with early EN, not reported in guidelines. There is room for error in retrospective data collection. Concerns regarding patient hemodynamic stability may have been overlooked during this process. Nursing staff may not be able to influence decisions made by the surgeon or consultant, but being alert to current evidence may aid them in questioning the rationale behind these decisions.

### 6.2 Patient's physiological condition and stability

The physiological condition of the patient and the nature of their surgery may affect their nutrition management. Guidelines promote early EN following surgery including that which involves the oesophagus and abdomen.^5^, ^7^, ^10^ However, complications following surgery directly to the GI tract have been reported including risk of anastomotic leak and ileus.^29^ ESPEN guidelines and The Enhanced Recovery After Surgery Society's guidelines for oesophagectomy patients recommend the use of early EN via feeding routes, such as NJ tubes, distal to upper GI surgical sites to help avoid leakage.^30^, ^31^ Severe complications involving this route have been reported, however, and the ideal route of administration still remains unclear.^30^, ^32^ This inconclusive evidence may be a reason why the surgical teams in this service evaluation did not choose to initiate early EN via this route following upper GI surgery.

### 6.3 MDT communication and guidance on EN administration

A delay in verbal or written communication regarding the nutrition plan or enteral tube position confirmation following radiological imaging, was found to cause delays in EN initiation. This leads to implementation of alternative plans as seen in 11% (*n* = 7) of all cases (*n* = 64) who were administered 50% glucose infusion on ICU admission. Improvements in communication between teams, for example, through person‐to‐person discussion on interdisciplinary patient care rounds may help reinforce information and reduce delays in EN initiation.^33^

Studies have shown that patients who receive dietician management reach nutritional goals quicker with improved outcomes.^33^ Issues commonly arise in their absence. Tignanelli et al.^34^ found the utilization of nutrition teams, made up of a dietician, a specialist pharmacist and specialist nutrition nurse, significantly lowered patient mortality rates (*p* < .001).

### 6.4 GI intolerance assessment protocols and continuous EN administration

Interrupting EN feed accounts for energy and protein targets not being met. In the data analysed, GRVs less than the recommended feed discontinuation threshold caused interruptions with the second longest median length of time (16 h; IQR: 4–30.5 h). The SCCM/ASPEN (2016), ESCIM (2017) and ESPEN (2018) guidelines all recommend a GRV threshold of ≥500 mL/6 h.^5^, ^7^, ^10^ The local enteral feeding policy in this centre recommend a lower threshold of GRVs of ≥500 mLs/4 h should prompt feed cessation. Despite this, interruption occurred when volumes as little as <200 mLs were obtained when no other signs of feed intolerance were recorded. The focus group reported methods of measuring and discarding GRVs vary between nurses, indicating poor knowledge of guidelines. The use of GRVs as a tool for recognizing GI intolerance is commonly used but evidence for its use is limited.^35^, ^36^ A uniform definition of enteral feeding intolerance is not present throughout the literature, which may contribute to feed being interrupted unnecessarily, and is required to improve practice.^37^

Additional observations for signs of gastrointestinal intolerance including abdominal discomfort, pain or distension, signs of nausea, vomiting, diarrhoea or constipation are recommended by guidelines.^33^ Any evidence of these issues found in the patient documentation were recorded as reasons for feed interruption. However, if documentation was not accurate, it may appear the feed was unjustifiably interrupted, skewing the results. Training nursing staff to conduct thorough GI assessments is paramount in protecting patients from adverse events when GRV measuring is unreliable.^5^ Local guidelines on GRV use should be adjusted to align with current recommendations. This conflict in guidance may have led to inaccuracies in the data obtained.

## 7 LIMITATIONS

This is a small‐scale preliminary study, so any conclusions must be taken with caution. The main limitation of this study was the small sample size (*n* = 64) included in the retrospective data analysis meaning it was prone to over estimation of any association found.^20^ Increasing the number of cases included would increase reliability. Patients intubated within the first 24 h were included to ensure they were reliant on artificial feeding and there was opportunity for decisions around EN feeding to be made; however, in retrospect, extending this timeframe to include patients intubated within the first 48 h of admission would have prevented exclusion of appropriate patients. The nutrition management practices were examined in a single‐centre only. Nutritional policies and practices vary between centres; therefore, the results cannot be generalized. There is a risk of recollection bias. The screening process involved relying on HCPs' documentation to gain insight into practice. Documentation may not be accurate, leading to incorrect assumptions and bias. Detailed notes were kept on each record as evidence of the time spent scrutinizing the data.

Data from only a single focus group were analysed because of inability to recruit a sufficient number of participants. This may have been because of several reasons including clinical workload demand or inconvenience. The moderator can help to improve nurses' participation in focus groups by providing face‐to‐face teaching on the study prior to recruitment rather than relying on to an email.^38^ Voluntary participation may have constituted self‐selection bias. Nurses who were not engaged in nutrition management were less likely to be involved.^39^ The focus group did include nurses with a range of experience and expertise, but it is insufficient to represent views from the whole unit, impacting the transferability and generalisability of results. In future, multiple focus groups would help ensure a variety of views are reported.

The author is an HCP on the unit; therefore, maintaining objectivity was difficult. The use of a third person to make field notes during the focus group discussion provided further objectivity.^40^ Numbers were used to identify participants during the transcription process to maintain confidentiality and reduce bias during analysis. The framework method used to analyse data displays transparent data analysis contributing to the validity of the study.

## 8 RECOMMENDATIONS

Ensuring nutrition management is regarded as a priority in ICU through training and education is a key factor which may help to improve practice. Promoting communication between MDT members may prevent delays in EN initiation. Clearer nutritional management recommendations set out by guidelines may reduce ambiguity around best practice. A larger service evaluation incorporating the views of a wider range of MDT members, especially surgical team members, would be beneficial to find out why certain feeding plans were chosen.

## 9 CONCLUSIONS

The results from the data analysis found the guidelines set out by ESPEN (2018), ESCIM (2017) and SCCM/ASPEN (2016) regarding EN delivery, were not being followed to the extent to which they were intended. A nurse focus group discussion provided a deeper insight into issues with nutritional management practice from a nursing perspective.

Critical analysis of these findings uncovered factors which influence guideline adherence in practice. Ensuring nutrition management is regarded as a priority in ICU through training, and education is a key factor which may help to improve practice. Promoting communication between MDT members may prevent delays in EN initiation. Clearer nutritional management recommendations set out by guidelines may reduce ambiguity around best practice. A further study with a greater number of cases incorporating views from the wider range of MDT members would be beneficial to determine the validity of these findings.

## CONFLICT OF INTEREST STATEMENT

The authors declare no conflicts of interest.

## ETHICS STATEMENT

This study was approved by the Committee of Clinical Research at The Royal Marsden NHS Foundation Trust. This study was NOT considered Research by the Health Research Authority.

## INFORMED CONSENT

Written consent was gained from the nursing staff involved in the focus group to use their data as part of the study.

## Data Availability

The data that support the findings of this study are openly available in UK Data Service, ReShare at https://dx.doi.org/10.5255/UKDA-SN-856513.
